# Nanotopography and oral bacterial adhesion on titanium surfaces: *in vitro* and *in vivo* studies

**DOI:** 10.1590/1807-3107bor-2024.vol38.0021

**Published:** 2024-03-11

**Authors:** Humberto Osvaldo SCHWARTZ-FILHO, Tauane Ramaldes MARTINS, Paulo Roberto SANO, Marcela Takemoto ARAÚJO, Daniel Cheuk Hong CHAN, Nathália Ramaldes SALDANHA, Kátia de Pádua SILVA, Talita Signoreti GRAZIANO, William Cunha BRANDT, Caio Vinícius Roman TORRES, Karina COGO-MÜLLER

**Affiliations:** (a) Universidade Federal do Paraná – UFPR, School, Department of Stomatology, Curitiba, PR, Brazil.; (b) Universidade de Santo Amaro – Unisa, Department of Dentistry, São Paulo, SP, Brazil.; (c) Universidade Estadual de Campinas – Unicamp, Piracicaba Dental School, Department of Physiological Sciences, Piracicaba, SP, Brazil.; (d) Universidade Estadual de Campinas – Unicamp, School of Pharmaceutical Sciences, Laboratory of Antimicrobial Pharmacology and Microbiology, Campinas, SP, Brazil

**Keywords:** Titanium, Biofilms, Dental Implants

## Abstract

The present study aimed to evaluate the influence of titanium surface nanotopography on the initial bacterial adhesion process by *in vivo* and in vitro study models. Titanium disks were produced and characterized according to their surface topography: machined (Ti-M), microtopography (Ti-Micro), and nanotopography (Ti-Nano). For the *in vivo* study, 18 subjects wore oral acrylic splints containing 2 disks from each group for 24 h (n = 36). After this period, the disks were removed from the splints and evaluated by microbial culture method, scanning electron microscopy (SEM), and qPCR for quantification of Streptococcus oralis, *Actinomyces naeslundii, Fusobacterium nucleatum*, as well as total bacteria. For the in vitro study, adhesion tests were performed with the species *S. oralis* and *A. naeslundii* for 24 h. Data were compared by ANOVA, with Tukey’s post-test. Regarding the *in vivo* study, both the total aerobic and total anaerobic bacteria counts were similar among groups (p > 0.05). In qPCR, there was no difference among groups of bacteria adhered to the disks (p > 0.05), except for *A. naeslundii*, which was found in lower proportions in the Ti-Nano group (p < 0.05). In the SEM analysis, the groups had a similar bacterial distribution, with a predominance of cocci and few bacilli. In the in vitro study, there was no difference in the adhesion profile for *S. oralis* and *A. naeslundii* after 24 h of biofilm formation (p > 0.05). Thus, we conclude that micro- and nanotopography do not affect bacterial adhesion, considering an initial period of biofilm formation.

## Introduction

Long-term oral rehabilitation using implants is considered a predictable technique with high success rates in fully and partially edentulous patients.^1^ Modifications of implant surfaces at the micro- and nanometric levels are factors that positively affect the osseointegration process and consequently the implant success rate.^2,3^ Surface treatments carried out by physical, chemical, and deposition methods can alter chemical characteristics, wettability, roughness, surface free energy, and topography, which can further influence cell adhesion to the implant surface.^4,5^

Complications due to bacterial adherence and colonization can occur after implant surgery, leading to inflammation of the mucosal tissue, bone loss, and in some cases, implant failure. The prevalence of such complications is highly inconsistent, varying according to patients’ conditions, diagnosis methods, and study variability;^6^ however, the prevalence of peri-implantitis is expected to range from 9.6% to 30% at the implant level and from 18% to 43% at the subject level, and the prevalence of mucositis is expected to be around 30% at the implant level and from 46% to 53% at the subject level.^6^

Theoretically, the surface of the implant inserted in the bone should not be exposed to the oral cavity after surgery and the healing period. However, in some clinical situations, the most coronal part of the implant surface may be exposed due to crestal bone remodeling during the postoperative healing period.^7^ Hence, bacteria could colonize these regions, causing mucositis and peri-implantitis. Among the main factors that can influence microbial adhesion to these exposed surfaces are roughness and topography.^8-13^ Nevertheless, researchers have shown that roughness does not increase the incidence of peri-implantitis.^14^

Changes in the surface produce structures that are measured in the scales of mm, μm, and nm. The extent to which these changes influence bacterial adhesion is limited to implant design (at the mm scale) and to surface roughness (at the μm scale). It is still unclear whether nanoscale surface topography can interfere with bacterial adhesion. Some nanometric surfaces have antibacterial properties that reduce bacterial colonization,^9,13,15-17^ whereas other surfaces promote bacterial attachment.^9^

Considering that there is no consensus on the influence of topography on microbial adhesion, we aimed to compare initial biofilm formation on three types of surfaces: machined, with micrometric scale (microtopography), and with nanometer scale (nanotopography). We performed an *in vivo* and in vitro study of biofilm formation on titanium disks and compared adherence using cell viability, quantitative polymerase chain reaction (qPCR), and scanning electron microscopy methods (SEM). We previously characterized the tested surfaces, and found promising results for the nanoscale surface regarding its potential to stimulate bone activity.^3^

## Methodology

### Experimental design

The *in vivo* study was designed following the appropriate recommendations of CONSORT.^18^ The in vitro study was planned and performed according to the CRIS guidelines.^19^ For the *in vivo* study, 18 volunteers wore oral acrylic splints (AS) that contained titanium disks for 24 h. After this period, biofilm formation was quantified by total microbial count of aerobes and anaerobes, according to the culture technique, and by qPCR, for quantification of total bacteria, Streptococcus oralis, *Actinomyces naeslundii*, and *Fusobacterium nucleatum*; bacteria were qualified by SEM. For the in vitro study, *S. oralis* and *A. naeslundii* adhesion on the surface of the disks was evaluated by counting the viable bacteria.

### Titanium specimens and surface characterization

Titanium specimens were manufactured and provided by the NEODENT (Curitiba, Paraná, Brazil). Three types of titanium surfaces were analyzed: Ti-M – machined; Ti-Micro – microtopography; and Ti-Nano – nanotopography. Samples of commercially pure titanium (grade 4) were machined into disks (10 mm x 2 mm) and subjected to a surface modification process. Microtopography was obtained by blasting with aluminum oxide particles, followed by acid conditioning (commercial grade). Nanotopography was obtained by treating with equal volumes of a solution of 30% H_2_SO_4_ and H_2_O_2_.^20^

Roughness, chemical composition, and morphology characterization of these titanium disks was carried out in a previous study.^3^ Topography characterization was performed at micrometric level using an optical interferometer (MicroXam; ADE Phase Shift Technology Inc., Tucson, USA), and at nanometric level by atomic force microscopy (Dimension 3000 SPMTM, Digital Instruments, Santa Barbara, USA). For the evaluation of the surface chemistry, X-ray dispersive energy spectroscopy (EDX; LEO 440 – Zeiss, Oberkochen, German) was carried out.

### *In vivo* study: bacterial adhesion on Ti disks in healthy patients

This study was carried out according to the principles of the Declaration of Helsinki.^21^ After receiving ethical approval from the University Santo Amaro (protocol no. 544.111), all subjects provided a written informed consent.

The sample size calculation was based on a previous study that was similar in nature.^22^ The selected outcome variable was the number of colony-forming units adhered to the titanium disks (log CFU/disks), both with and without surface treatment. To establish the number of individuals to be included in the present study, a sample size calculation (CA) was performed using the statistical formula: MSD = t5% √2*DMe/N; where, MSD is the minimum significant difference that one would want to observe for each variable of interest, t(5%), a tabulated value of 2, and DMe is the observed dispersion measure, and N is the number of patients. Assuming a safety margin of 10%, it was calculated the number of 18 patients to be included in this study.

Eighteen adult volunteers aged 18 to 40 years were selected to participate in the study. The selected patients were systemically healthy (no endocrine, hematological, or autoimmune disorders, no nutritional changes, no diseases or consumption of drugs that alter salivary flow), nonsmokers, and with excellent oral conditions (lack of carious lesions and periodontally healthy). Plaque index and periodontal probing were performed by an experienced periodontist. Only patients with periodontal health were selected, with no history of treatment and no clinical signs of gingivitis and periodontitis, no insertion loss, probing depth of up to 3 mm, bleeding on probing in less than 10% of sites, and no radiographic bone loss.^23^ The patients had a salivary flow of 1.2 ± 0.2 mL per minute and had no changes in salivary glands. Exclusion criteria were the use of antimicrobials, mouthwashes, and nicotine consumption in the three months prior to the study, use of orthodontic appliances, pregnancy, and lactation.^8,11^

**Figure 1:**
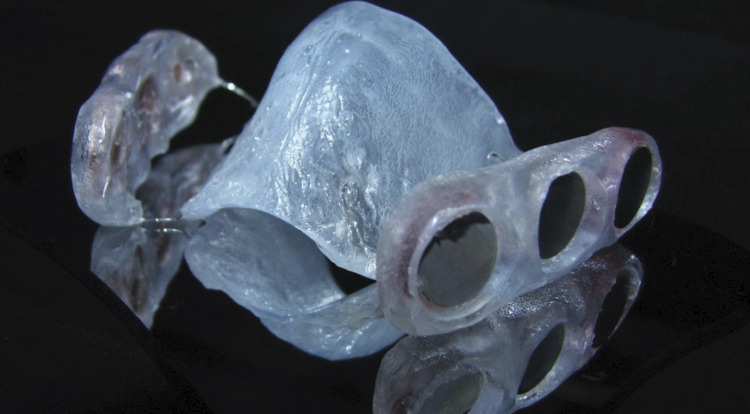
Acrylic splint (AS) containing titanium disks allocated in the niches prepared on the buccal areas.

For the *in vivo* bacterial adhesion assay, AS was manufactured as previously described.^22^ Two disks of each of the three titanium surfaces (Ti-M, Ti-Micro, and Ti-Nano) were fixed in the premolar and molar region of both buccal sides of the splint, as demonstrated in Figure 1. The disks were fixed with light-cured resin (Filtek™ P60, 3M, Espe, USA). Prior to use, the splint was submitted to a disinfection protocol: debridement in an ultrasound bath for 15 minutes, followed by immersion in 1% sodium hypochlorite solution for 15 minutes for chemical disinfection. Thereafter, the set was washed 3 times with distilled water to remove excess solution.^22,24^

Patients were instructed to wear the AS in the upper jaw for 24 h and to remove it only at mealtime and for tooth brushing, when it remained at 100% humidity (in a humidifying container with distilled water and sterile gauze to maintain the biofilm). During the period that volunteers were to use the splint, they were instructed to maintain their eating habits and routine oral hygiene, using the oral hygiene material provided by the researchers.^24^ In addition, they were instructed not to brush the region of the splint where the disks were placed.

After 24 h, a total of 36 disks (per group) were carefully removed from the splints and part of them were placed in sterile polystyrene tubes containing 5-mL sterile PBS buffer (n = 30/group). These disks were vortexed for 1 min and sonicated for 1 min at 5% amplitude with 6 pulses, 9.9 s per pulse and 5 s interval (Vibra-Cell™ Ultrasonic Liquid Processor, Newtown, USA) to detach and suspend the biofilm in the PBS medium. Then, an aliquot was used for microbiological culture analysis and the remainder was frozen at -80 °C for later analysis by qPCR. For SEM analysis, the disks (n = 6/group) were treated separately.

### Quantification of microorganisms adhered to the disk by the culture technique

The bacterial culture technique was used to quantify viable microorganisms (aerobic and anaerobic) adhered to the different surfaces. Schaedler Blood Agar (SBA- Difco^®^, Detroit, USA), added with 5 µg/mL of hemin, 1 µg/mL menadione, and 5% of sheep blood, was used to cultivate anaerobic bacteria, whereas Tryptic Soy Agar (TSA-Difco^®^) culture medium was used to grow aerobic bacteria. With the bacterial suspension obtained from the removal of the biofilm from the disks, serial dilutions were made (10 to 10,000 times) and 10 µL of each dilution were plated in both Schaedler and TSA agars.

SBA plates were incubated in anaerobic jars containing an anaerobic atmosphere generation system – Anaerobac (Probac, São Paulo, Brazil). The jars were placed in an incubator for 7 days, whereas TSA plates were incubated for 5 days, both at 37°C. After the respective periods, colony count was performed to determine the colony-forming units per mL (CFU /disk).

### Quantitative PCR – qPCR

Quantification of *Actinomyces naeslundii, S. oralis*, and F. nucleatum, and total bacteria in biofilm adhered to the titanium disks was performed by qPCR technique. Primer sequences are described in Table. These primers were previously designed using the Primer3 tool (http://www.bioinformatics.nl/cgi-bin/primer3plus/primer3plus.cgi) and checked to confirm target specificity using the BLAST tool (https://www.ncbi.nlm.nih.gov/tools/primer-blast/).

**Table:** Primer sequences used for detection of total bacteria and *A. naeslundii, S. oralis,* and *F. nucleatum* by the qPCR technique.

Species	Primer sequences (F – forward; R – Reverse)	References
Total quantification (Universal primers)	5’TGGAGCATGTGGTTTAATTCGA3’	Nonnenmacher et al., 2004^23^
	5’TGCGGGACTTAACCCAACA3’	
*A. naeslundii*	5’GTCTCAGTTCGGATCGGTGT3’	Designed from the gene sequence 16SrRNA
	5’CCGGTACGGCTACCTTGTTA3’	
*S. oralis*	5’TTGGCTCAATTCCCTTTGAC3’	Designed from the gene sequence *rgg*
	5’GTCCAAACAAGCCACCACTT3’	
*F. nucleatum*	5’CGCAGAAGGTGAAAGTCCTGTAT3’	Kato et al., 2005^24^
	5’TGGTCCTCACTGATTCACACAGA3’	

DNA extraction was performed using the PureLink™ Genomic DNA Mini Kit (Invitrogen – Thermo Fisher, Carlsbad, USA) according to the manufacturer’s recommendations, with minor modifications. The incubation time of the lysis step (incubation with Proteinase K and PureLink™ Genomic Lysis/Biding Buffer) was changed to 2 h. For biofilm samples of titanium disks, Gram-positive bacteria extraction methods were used, and for DNA extraction from pure bacterial strains, the Gram-positive or negative extraction method was used according to each bacterium.

For standard curve construction, the genomic DNA (gDNA) extracted from pure cultures of Escherichia coli ATCC 10536 (total quantification), *A. naeslundii* ATCC 12104, *S. oralis* ATCC 10557, and F. nucleatum ATCC 51190 was used. Standard curves with gDNA concentrations between 10^8^and 10 copies were constructed, representing the bacterial cell number.^25^

qPCR reaction was carried out in a total volume of 8 μL, containing 5 μL of SYBR Select Master Mix (Thermo Fisher Scientific, Waltham, USA), 2 μL of DNA template, and 1 μL of primer pair solution (300 nM/reaction). qPCR reactions were performed in the StepOne^®^ real-time thermocycler (Applied Biosystems^®^ Thermo Fisher Scientific, Waltham, USA). The initial conditions of the cycles were: 50°C for 2 min (UDG incubation); 95°C for 2 min; followed by 40 cycles of 95°C for 15 s; and 60°C for 30 s. Dissociation curves were constructed after the end of the cycles in order to confirm the specificity of the PCR products. For bacterial quantification, the absolute quantification method was used by comparing the threshold cycle (Ct) of the samples with the Ct of the bacterial curves.^25^

### Qualitative analysis of bacterial adhesion by SEM

Biofilm formed in the surface of the disks were fixed at 3 mL of PBS solution with 3% glutaraldehyde for 24 h at room temperature. After this period, the solution was removed and the disks were dehydrated in increasing concentrations of ethanol (60%, 70%, 80%, 90%, and absolute alcohol), remaining for 5 min in each concentration. The disks were then dried at room temperature and coated with gold-palladium in a 50 mA metallizer for 3 min. Samples were analyzed using SEM (JSM 5600LV model, Jeol, Tokyo, Japan) with a magnification of 1,200, 2,500 and 4,000 times.

### *In vitro* study – bacterial adhesion of *S. oralis* and *A. naeslundii* on Ti disks

Streptococcus oralis ATCC 35037 and *Actinomyces naeslundii* ATCC 12104 were used for the initial biofilm formation of mono-species. *S. oralis* was routinely grown on Brain Heart Infusion agar (BHI – Difco™) in a 5% CO_2_ atmosphere, at a temperature of 37°C. *A. naeslundii* was grown on BHI agar added with 5 µg/mL of hemin, 1 µg/mL of menadione, and 1g/L of cysteine, at 37 °C, under anaerobic conditions (10% CO_2_, 10% H_2_, and 80% N_2_ – MiniMacs Anaerobic Workstation, Don Whitley Scientific, Shipley, UK). For the bacterial adhesion assay, the culture medium suggested by Sánchez et al. (2011)^26^ was used (BHI 37 g/L; mucin 2.5g /L; cysteine 1g/L; sodium bicarbonate 2 g/L; yeast extract 1g/L; hemin 5 µg/mL; and menadione 1 µg/mL), referred to here as modified BHI.

To prepare the bacterial inoculum, cultures on the BHI agar medium were incubated for 24 h. From these cultures, two to three colonies of *S. oralis* or *A. naeslundii* were collected and suspended in modified BHI broth without mucin. The inoculum was adjusted to an absorbance value of 0.1 (wavelength – 660 nm; approximately 1.0 – 2.0 x 10^8^ CFU /disk).

Mono-species biofilms were formed on titanium disks coated with clarified and filter-sterilized whole human saliva as previously described.^27,28^ The saliva collection from one donor was approved by the Research Ethics Committee of the Piracicaba Dental School, University of Campinas (Protocol # 56790616.4.0000.5418). Sterile disks were vertically anchored in metallic devices and placed in the 24-well plate containing 3 mL of the filtered saliva solution. Disks were incubated in the orbital shaker at 37°C for 2 h at 60 rpm. Then, disks were removed from the saliva solution in which the acquired film was formed. The saliva-coated titanium disks were vertically placed in batch cultures of *S. oralis* or *A. naeslundii* containing 2.7 mL of modified BHI medium and 0.3 mL of bacterial inoculum. Disks were incubated for 24 h to allow the initial establishment of biofilms. Biofilm assays were carried out in quintuplicate in at least three different experiments.

After incubation, disks were removed from the plates and gently washed in sterile 0.9% NaCl solution to remove weakly-adhered bacteria. Then, they were transferred to a polystyrene tube containing 5 mL of sterile saline solution, vortexed for 1 minute, and sonicated for 1 minute with 5% amplitude and 6 pulses, 9.9 s each pulse and 5 s interval (Vibra-Cell™ Ultrasonic Liquid Processor). This bacterial suspension was serially diluted, and 10 µL of each dilution was seeded on BHI Agar. After completion of 48-h incubation, colonies were counted to determine CFU/mL

### Statistical analyses

The data were tested to determine their distribution (parametric or non-parametric) using the Shapiro-Wilk test. Microbiological data (log CFU/disk) were compared among the groups (Ti-M, Ti-Micro, and Ti-Nano) using ANOVA and Tukey’s test. The significance level was set at 5%. The analyses were performed using GraphPad Prism version 8.0 (San Diego, USA).

## Results

### *In vivo* study

**Figure 2:**
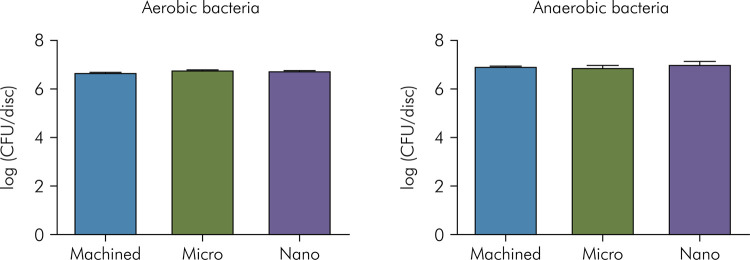
Logarithmic mean and standard deviation for aerobic and anaerobic viable bacteria per disk according to cultures (*in vivo* study). No differences were observed among the groups (ANOVA).

The aerobic and anaerobic quantification of viable bacteria were performed after culturing samples of biofilm detached from titanium disks. Logarithmic data of aerobic and anaerobic colony forming units is shown in Figure 2.

The logarithmic means and standard deviations of the aerobic count for the Ti-M, Ti-Micro, and Ti-Nano disks were 6.65 ± 0.38, 6.72 ± 0.32, and 6.70 ± 0.44, respectively. For anaerobic counts, the logarithmic means and standard deviations for the Ti-M, Ti-Micro, and Ti-Nano disks were 6.86±0.27, 6.80±0.50, and 6.94 ± 0.41. In both the aerobic quantification (p=0.848) and anaerobic quantification (p = 0.6703), there was no difference between the machined, microtopography and nanotopography groups, i.e., there was no increase in the number of bacteria due to disk surface topography (p > 0.05, ANOVA).

**Figure 3:**
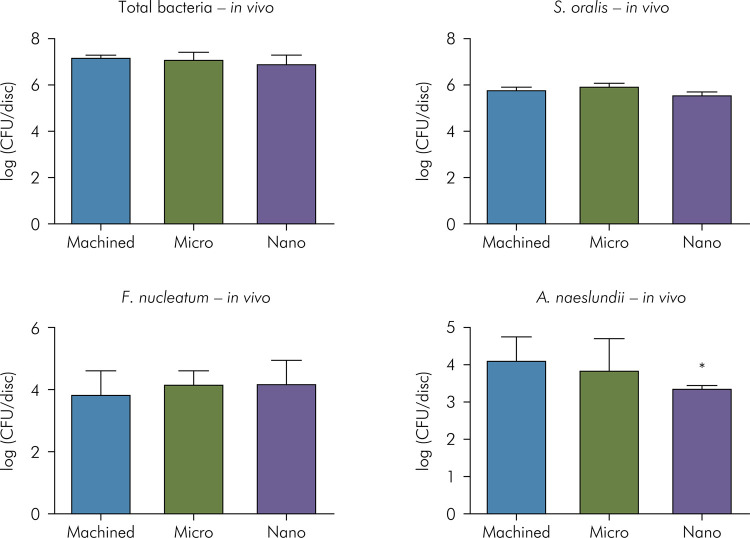
Mean and standard deviation of total bacteria, *S. oralis, F. nucleatum*, and *A. naeslundii* of disk samples by qPCR (*in vivo* study). No differences were observed for total bacteria, *S. oralis,* and *F. nucleatum* (p > 0.05, ANOVA). *A. naeslundii* showed lower adhesion to the nanoscale roughness disks compared with machined and microscale surfaces (p < 0.05, ANOVA, Tukey’s test).

The samples were also evaluated for total bacterial levels and specific bacterial quantification for Streptococcus oralis, *Actinomyces naeslundii*, and *Fusobacterium nucleatum*. The initial adhesion of these species to the titanium surface were comparable among groups (p > 0.05, ANOVA), except for *A. naeslundii*, that were in lower levels in nano-scale topography implant surface (p<0.05, ANOVA, Tukey). Figure 3 shows logarithm cell number for the total surface area of the disk.

**Figure 4:**
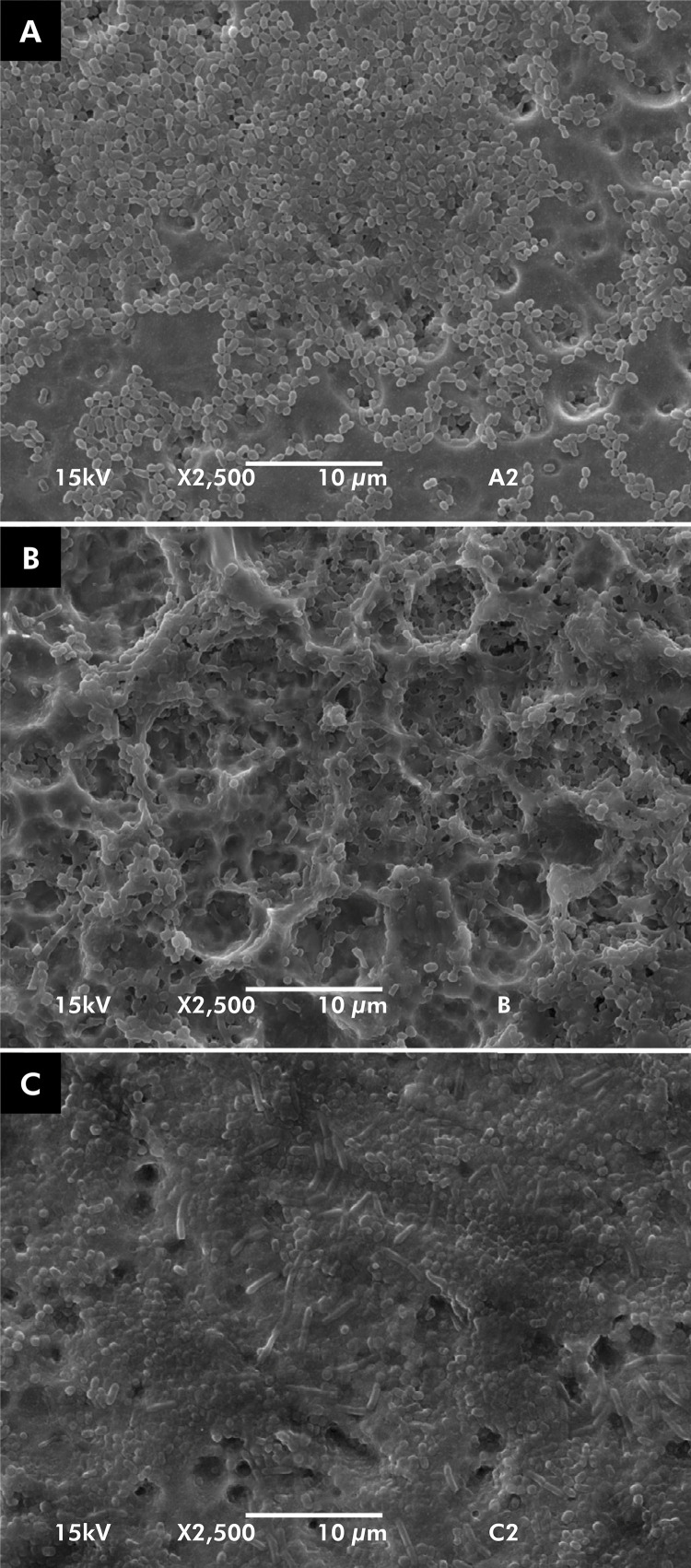
Scanning electron microscopy (2.500x) for Ti-M (1), Ti-Micro (2), and Ti-Nano (3) after *in vivo* experiments.

Additionally, SEM was performed to visualize the adhesion of microorganisms on the disk surface. The images are shown at 2500x magnification for micro-scale topography, nano-scale topography, and machined disks (Figure 4). It was possible to observe the adhesion of microorganisms uniformly distributed on the entire surface of the three experimental groups. Cocci predominated in all samples, with a small representation of bacilli attached to the surfaces. Moreover, corroborating our findings for bacterial quantification, we observed that the bacterial colonization was similar among the implant surfaces.

### *In vitro* study

**Figure 5:**
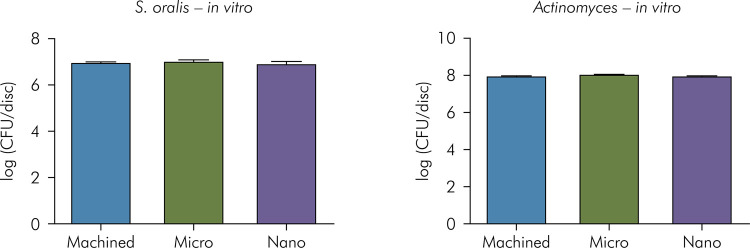
Logarithmic mean and standard deviation of colony-forming units of the initial biofilm formation for *S. oralis* and *A. naeslundii* (*in vitro* study). No differences were observed among the groups (ANOVA).

In order to verify the pattern of initial adhesion of first colonizers on different topographies, *S. oralis* and *A. naeslundii* mono-species biofilms were tested. Bacteria were grown on titanium surfaces for 24 h and total cell count was performed after disruption of biofilm adhered to the disks. Figure 5 shows the CFU/disk logarithm for the 3 surfaces tested. Adhesion was similar for both species, regardless of the surface topography (p > 0.05, ANOVA).

## Discussion

Implant surfaces have been modified to increase the bone contact area, thereby improving osseointegration.^29^ However, it is still controversial whether and to what extent topography can interfere with microbial adhesion. In our study, we compared three titanium surfaces, previously characterized^3^ and referred to here as machined (Ti-M), microtopography (Ti-micro) and nanotopography (Ti-nano) surfaces, in terms of biofilm formation and bacterial adhesion. Topographic features in the titanium surface did not affect biofilm formation in vitro and in vivo.

The studied surfaces were previously characterized for chemical composition, roughness, and topography, and the results were published elsewhere.^3^ The Ti-Micro surface (previously named “BAE”) showed higher roughness (mean and standard deviation of Sa = 0.57 ± 0.05 µm) than the Ti-Nano (previously named “ON”) (Sa = 0.27 ± 0.07) and the Ti-M (Machined) (Sa = 0.28 ± 0.02).^3^ The smooth Ti-M surface presents some grooves randomly distributed by the machining procedure, whereas the Ti-Micro (BAE) surface had crater-like microscale characteristics. In the Ti-Nano (ON) surface, a nanoscale pitted topography was observed. Chemical composition was kept similar after surface treatments.^3^ In addition, the previous study showed that nano-scale topography modulated cytokine production in fibroblast culture, thus favoring a less-inflammation prone scenario that can stimulate the osseointegration process.^3^

Although knowledge of the bacterial adhesion process has expanded, how and to what extent surface topography influence bacterial attachment is not yet fully elucidated.^13^ In the present study, bacterial adhesion was examined for a 24-h period, representing the initial biofilm formation. According to the results found in microbial quantification by culture technique, SEM, and qPCR, the bacterial colonization profile was not different between the surfaces, except for *A. naeslundii*, which exhibited lower colonization of the surface with nanoscale topography in vivo. However, in vitro, *A. naeslundii* and *S. oralis* showed the same colonization pattern regardless of the surface. Some studies considered that surfaces with nanoscale topography or rougher surfaces can increase bacterial adhesion,^9^ whereas others argue that they have a certain anti-adhesive property.^13,16,17^ Nevertheless, according to both in vitro and in vivo studies, the nano- or microscale topographic features may not substantially interfere in bacterial adhesion and biofilm formation.

It is worth differing topography from roughness parameters. Roughness represents the variation in height of a surface, and topography describes the three-dimensional aspects characterized by shape and features in vertical spatial arrangement.^13,30^ The studied surfaces showed similar roughness with considerably different topography. Therefore, topography per se is a feature that is claimed to influence bacterial attachment to a surface, considering that several nanoscale surfaces have shown antimicrobial properties.^9,13,15,16^ These so-called nano-enabled mechanisms, such as physicochemical forces, cell membrane deformation, and chemical gradient, can repeal bacteria from the surface or even cause ruptures on the bacterial cell membrane.^13,31^ Furthermore, some researchers believe that the increase in surface roughness at the nanoscale level can cause an unfavorable condition for the adhesion of bacteria, since the bacteria size is at a microscale level.^32^ These hypothetical properties of nanosurfaces do not seem to substantially interfere in the bacterial attachment to the nanoscale topography surface (Ti-Nano). In fact, many researchers who aimed to test nanoscale surfaces evaluated different materials, such as alumina, gold, silica, among others, other than titanium,^15,16,33^ an important feature that can also interfere with bacterial adhesion. However, other studies performed with nanoscale titanium surface showed both reduction^17^ and increase^9^ in bacterial attachment.

Interaction with nanoscale surfaces can impact bacterial morphological features rather than biofilm formation. Previously, it was demonstrated that a nanoscale gold surface did not interfere with the level of bacterial attachment, but reduced the expression of type I fimbriae and enhanced cpxP and degP genes, which are involved in the envelope stress response by E. coli.^33^ In our study, we did not evaluate genotypic and phenotypic features of the biofilm; however, if these changes occurred during titanium interaction, they did not affect biofilm formation. Another fact that may interfere with bacterial adhesion to nanoscale surfaces is pore size. Alumina surfaces with cylindrical nanopores of 15 and 25 nm reduced bacterial attachment compared with 50 and 100 nm pores. The nanoscale surface (Ti-Nano) showed pores of around 270 nm, and it did not have antimicrobial properties probably due to the higher roughness. Besides, surface charge, conditioning film, and protein adsorption, among others, are examples of other parameters that can affect bacterial adhesion to a surface.^13,32^ More studies comparing nanoscale topography with different roughness levels are necessary to understand this relationship.

It is not well established to which extent roughness can interfere with bacterial adhesion.^13^ The samples of the Ti-Nano and Ti-M groups were considered smooth surfaces, whereas Ti-Micro is characterized as a moderately-rough surface.^2^ Some previous studies have shown a direct relationship between increased roughness and microbial adhesion.^7,8,10-12,34^ However, other studies performed with surfaces with roughness lower than 1.0 micrometer^10,35^ or even with roughness higher than 1.0 micrometer^22,36^ contradicted the relationship between roughness and microbial adhesion. In a previous study, authors compared the in vivo biofilm formation on three different titanium surfaces, named Ti-M (machined) and Ti-AE acid-etched, with roughness of approximately 0.7 µm, and Ti-AL (anodized and laser irradiated) with roughness of around 1.4 µm. Despite the difference in roughness of around 0.7 µm, the total bacterial and *S. oralis* load in the titanium disks were very similar between the surfaces.^22^ We found surface roughness of around 200 nm in our study, except for the surface with microscale topography, which has an average roughness of 570 nm. Thus, we can suggest that surfaces with roughness below 500 nm, as in our study, do not seem to influence the initial bacterial adhesion.

There are many divergent data from in vitro and in vivo studies comparing the influence of surface characteristics on biofilm formation. In the present study, only the *A. naeslundii* adhesion was lower in the nanoscale topography surface (around 1.5 log lesser than Machined), with no differences for total bacterial counts or for F. nucleatum and *S. oralis* numbers. Many studies that stated that roughness and topography can alter bacterial adhesion consider changes of less than 1 log for bacterial assessment.^8,11,12^ Differences less than 1 log in the number of bacteria are irrelevant to biofilm formation.^22,37^ Therefore, such contradictory results may be partially explained by differences in the logarithmic ratio of bacteria adhered to a surface, among other factors involved in the study design.

Microscale topography and roughness are considered factors that can increase the adhesion, since they increase the surface area and pores on the surface can protect against oral shear forces.^7,34^ Surfaces with nano- and microscale topography showed pores, but such characteristics did not increase bacterial accumulation. In a study comparing different surfaces of the entire dental implant with microscale topography, the authors found bacteria sheltered in pores, with greater number of bacteria adhered to the moderately-rough surface compared with the minimal-rough one.^7^ In our study, we could not distinguish from SEM images whether bacteria were lodged into pores of micro and nanoscale topography surfaces. Previously, it was found that implant surfaces with microscale topography and roughness of approximately 700 nm (Ti-AE) and 1,400 nm (Ti-AL), with pores and grooves in their surfaces, did not influence the initial bacterial adhesion.^22^ In the present study, these surface-related features did not alter bacterial composition and adhesion.

Factors, such as roughness, wettability, and topography, are believed to have less influence in more advanced stages of biofilm maturation than in the early stages.^38^ Some studies showed that in the earliest stages of biofilm formation, surface properties may influence the quality and amount of biofilm formed.^10,35,38^ Other authors showed that such interference can occur even in more mature stages.^7,39^ We used in vivo and in vitro models of early biofilm formation (24 h) and, overall, no differences were observed. According to SEM images, we found a predominant monolayer biofilm, with many cocci and few bacilli, which typify an early stage biofilm. Therefore, titanium surface characteristics did not substantially interfere in early biofilm formation. Corroborating our findings, authors of previous studies showed that roughness and topography did not affect biofilm formation in early stages.^22,37^ It is believed that in vitro models are more susceptible to surface-related characteristics than in vivo models. Although competitive, the conditions in vivo are more abundant, and factors such as the microorganism’s variability, nutrient availability, and host conditions may compensate surface-related characteristics when forming and maturing the biofilm.^38^

In the present study, the evaluation was conducted on biofilm-initiating microorganisms such as *S. oralis* and *A. naeslundii*, as well as later colonizer microorganisms like F. nucleatum. Furthermore, levels of P. gingivalis, a late colonizer and periodontal pathogen, were assessed in the samples but remained unidentified (data not shown). In biofilm formed in patients with good oral health, there is a higher prevalence of early colonizers (predominantly Gram-positive cocci) in the initial stage of the biofilm, compared to late colonizers (Gram-negative bacilli).^40,41^ Late colonizers are more commonly found in samples from patients with oral diseases, including periodontitis and peri-implantitis.^40,42^ Here, the bacterial adhesion occurred in healthy oral conditions. Future studies should examine the bacterial adhesion profile on titanium surfaces in oral disease conditions.

There is still a contradiction as to whether nanoscale topography can increase, reduce, or not alter microbial adhesion to surfaces. Many researchers point to an anti-adhesive activity for nanoscale surfaces,^13,16,17^ whereas others suggest an increase in microbial adhesion.^43^ In our study, we did not find these features for our microscale and nanoscale surfaces. The studied in vivo conditions were not as in the clinical implant situation, but they closely simulate clinical conditions. Moreover, there are no conclusive reports in the literature that rougher surfaces with micrometric or nanometric topography lead to greater implant loss or peri-implantitis.^14^ Thus, we believe that rough surfaces with nano- or micrometric topography, can stimulate osseointegration and be successful in implantation without causing a considerable risk for biofilm formation, infection, and subsequent loss of the dental implant. However, further studies are necessary to clarify the relationship between surface parameters and their interaction with microorganisms, including clinical studies.

## Conclusion

In summary, titanium topographies at the nano or microscale did not interfere with initial biofilm formation on titanium surfaces.
